# HDGF stimulates liver tumorigenesis by enhancing reactive oxygen species generation in mitochondria

**DOI:** 10.1016/j.jbc.2023.105335

**Published:** 2023-10-10

**Authors:** Tsung-Hui Hu, Jian-Ching Wu, Shih-Tsung Huang, Tian-Huei Chu, Ai-Jie Han, Ting-Wei Shih, Yi-Chen Chang, Shih-Ming Yang, Chou-Yuan Ko, Yu-Wei Lin, Mei-Lang Kung, Ming-Hong Tai

**Affiliations:** 1 Division of Hepato-Gastroenterology, Department of Internal Medicine, Kaohsiung Chang Gung Memorial Hospital, Chang Gung University College of Medicine, Kaohsiung, Taiwan; 2 Department of Internal Medicine, Kaohsiung Chang Gung Memorial Hospital, Chang Gung University College of Medicine, Kaohsiung, Taiwan; 3 Doctoral Degree Program in Marine Biotechnology, National Sun Yat-sen University, Kaohsiung, Taiwan; 4 Doctoral Degree Program in Marine Biotechnology, Academia Sinica, Taipei, Taiwan; 5 Medical Laboratory, Medical Education and Research Center, Kaohsiung Armed Forces General Hospital, Kaohsiung, Taiwan; 6 Institute of Medical Science and Technology, National Sun Yat-sen University, Kaohsiung, Taiwan; 7 Institute of Biomedical Sciences, National Sun Yat-sen University, Kaohsiung, Taiwan; 8 Department of Gastroenterology, Kaohsiung Armed Forces General Hospital, Kaohsiung, Taiwan; 9 Department of Radiation Oncology, Kaohsiung Veterans General Hospital, Kaohsiung, Taiwan; 10 Department of Medical Education and Research, Kaohsiung Veterans General Hospital, Kaohsiung, Taiwan; 11 Center for Neuroscience, National Sun Yat-sen University, Kaohsiung, Taiwan

**Keywords:** hepatoma-derived growth factor, ROS (reactive oxygen species), SOD2 (superoxide dismutase 2), mitochondrial energetics, nucleolin, NAC

## Abstract

Hepatoma-derived growth factor (HDGF) overexpression and uncontrolled reactive oxygen species (ROS) accumulation are involved in malignant transformation and poor prognosis in various types of cancer. However, the interplay between HDGF and ROS generation has not been elucidated in hepatocellular carcinoma. Here, we first analyzed the profile of HDGF expression and ROS production in newly generated orthotopic hepatomas by ultrasound-guided implantation. *In situ* superoxide detection showed that HDGF-overexpressing hepatomas had significantly elevated ROS levels compared with adjacent nontumor tissues. Consistently, liver tissues from HDGF-deficient mice exhibited lower ROS fluorescence than those from age- and sex-matched WT mice. ROS-detecting fluorescent dyes and flow cytometry revealed that recombinant HDGF (rHDGF) stimulated the production of superoxide anion, hydrogen peroxide, and mitochondrial ROS generation in cultured hepatoma cells in a dose-dependent manner. In contrast, the inactive Ser103Ala rHDGF mutant failed to promote ROS generation or oncogenic behaviors. Seahorse metabolic flux assays revealed that rHDGF dose dependently upregulated bioenergetics through enhanced basal and total oxygen consumption rate, extracellular acidification rate, and oxidative phosphorylation in hepatoma cells. Moreover, antioxidants of N-acetyl cysteine and MitoQ treatment significantly inhibited HDGF-mediated cell proliferation and invasive capacity. Genetic silencing of superoxide dismutase 2 augmented the HDGF-induced ROS generation and oncogenic behaviors of hepatoma cells. Finally, genetic knockdown nucleolin (NCL) and antibody neutralization of surface NCL, the HDGF receptor, abolished the HDGF-induced increase in ROS and mitochondrial energetics. In conclusion, this study has demonstrated for the first time that the HDGF/NCL signaling axis induces ROS generation by elevating ROS generation in mitochondria, thereby stimulating liver carcinogenesis.

Hepatocellular carcinoma (HCC) is the fourth leading cause of cancer-related death worldwide and ranks sixth in new cases globally (1). To date, its risk and pathogenesis have been extensively investigated, and approximately 80% of HCC is due to chronic infection with hepatitis B virus and hepatitis C virus (HCV); other causes of HCC are carcinogen stimuli, lipid accumulation, and heavy alcohol use (2). Hepatocarcinogenesis is initiated from cirrhosis and follows a series of events that are involved in different stages of the development of cirrhotic nodules, the development of early-stage HCC, and the progression of advanced HCC. HCC can be triggered by multiple factors, including intrinsic factors (such as genomic instability and gene mutation) and extrinsic factors (such as inflammation, oxidative stress, and microenvironmental remodeling), as well as molecular aberration-mediated signaling dysregulation (3, 4). Moreover, in this multistep carcinogenesis, chronic inflammation contributes to numerous complex pathogeneses that increase the levels of oxidative stress (5) and inflammatory cytokines, including transforming growth factors (TGFs), interleukins (ILs), and tumor necrosis factors (TNFs), in tumor cells to trigger oncogenic signaling pathways, ultimately resulting in hepatocarcinogenesis (3, 6).

Oxidative stress is always accompanied by the activation of undesirable signaling pathways, such as immune-related TGF-β- or TNF-induced signaling, and dysregulation of intracellular and/or mitochondrial antioxidant systems, which are closely correlated with the progression of HCC (7, 8). Once an aberrant or lost endogenous antioxidant network exists, the development of liver cancers becomes faster and more malignant as a carcinogen stimulus (9). Reactive oxygen species (ROS) are a group of highly unstable reactive ions and molecules and oxygen-derived free radicals, including superoxide anion (O_2_^–^), hydroxyl radical (^․^OH) and hydrogen peroxide (H_2_O_2_). ROS can be generated in extracellular and intracellular regions, including the mitochondrial electron transport chain (ETC), endoplasmic reticulum system, and NADPH oxidase (NOX) complex, and ROS in hepatocytes are primarily generated through mitochondrial metabolism, including mitochondrial bioenergetics and mitochondrial dynamics (10). ROS production also plays pivotal roles in a variety of normal biological conditions, such as proliferation, migration, and adhesion. In addition to intracellular ROS generation, a mitochondrial enzymatic scavenging system, which consists of Mn-dependent superoxide dismutase (MnSOD or SOD2), catalase, and glutathione peroxidase (GPX) and/or peroxiredoxins (PRX), is able to appropriately catalyze excess O_2_^–^ into H_2_O_2_ and then detoxify it into water and oxygen (11). Thus, uncontrolled oxidant generation (such as ROS and nitrogen species) induces ROS accumulation and oxidative stress, further disturbs cellular biological homeostasis and causes diverse pathophysiological conditions, such as genetic and epigenetic changes, to induce carcinogenesis (12) and cancer progression (13).

Hepatoma-derived growth factor (HDGF) is a mitogen originally purified from the conditioned medium of Huh-7 hepatoma cells. Moreover, our previous study demonstrated that HDGF was highly correlated with the pathogenesis of HCC and accordingly suggested that HDGF was a prognostic factor for HCC (14). We also found that HDGF was involved in various liver diseases, such as liver fibrogenesis (15) and concanavalin A-induced hepatitis (16). Indeed, HDGF has been demonstrated to play important roles in various pathological processes, including cancer cell growth, transformation, apoptosis, and metastasis. Therefore, studies have indicated that HDGF upregulation is found in numerous types of tumors, and its intensity and distribution in tumor cells are positively correlated with clinicopathological signatures (17). Several regulatory mechanisms of HDGF are associated tumor progression, including PI3K/AKT and ERK signaling pathway activation (17), podosome formation (18), epithelial-mesenchymal transition promotion (19, 20), and vascular endothelial growth factor (VEGF) induction (21). Recently, our group demonstrated that HDGF interacts with its receptor nucleolin (NCL) and triggers downstream PI3K/AKT signaling activation to promote liver carcinogenesis (22). However, the role of HDGF in the promotion of liver carcinogenesis is still not sufficiently understood. The present study aimed to elucidate the function and mechanism of HDGF in regulating redox homeostasis and mitochondrial bioenergetics during liver carcinogenesis.

## Results

### HDGF expression is correlated with ROS generation in hepatic tissues and HCC

**Figure 1:**
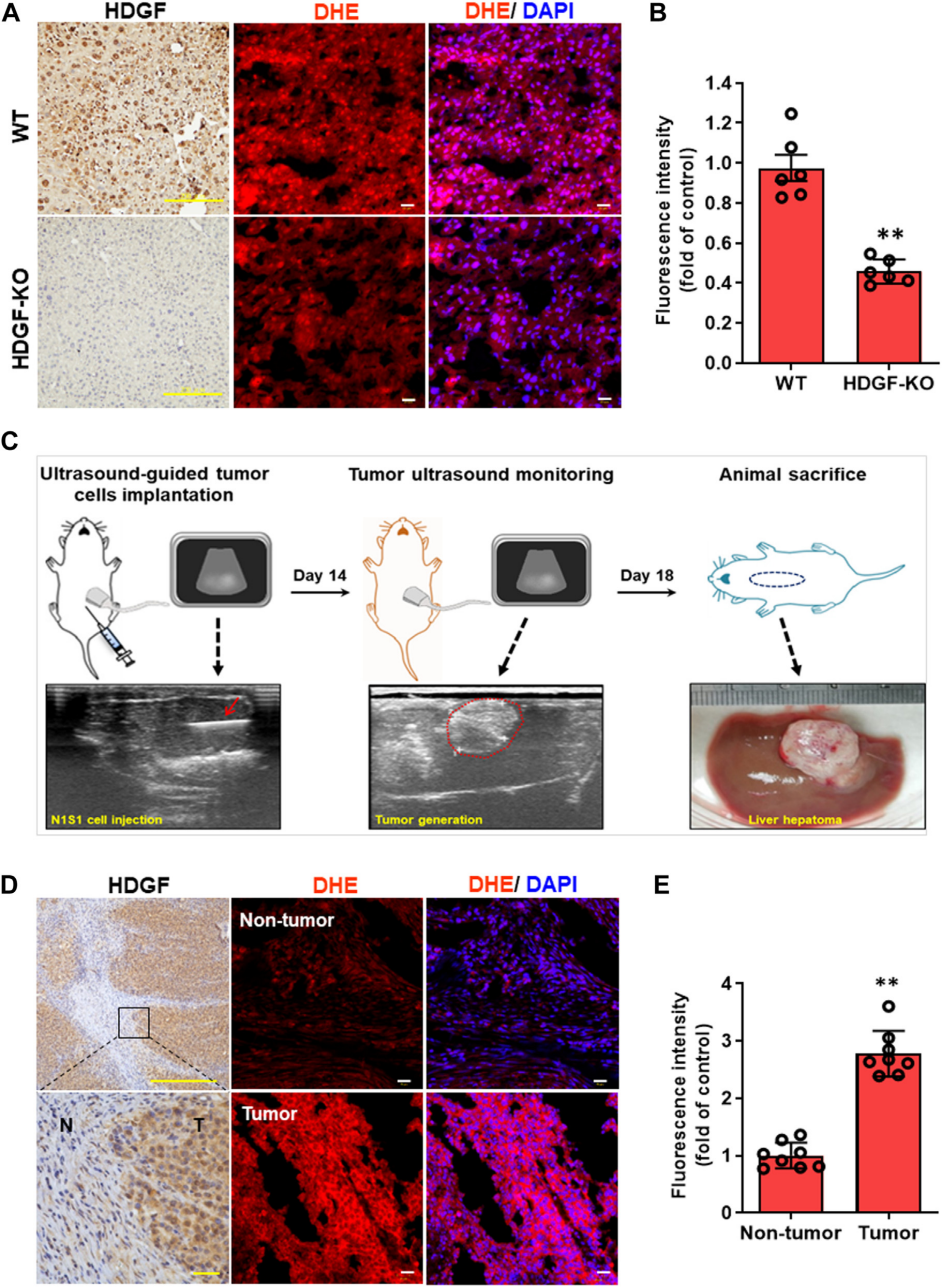
**Correlation between HDGF expression and ROS generation *in vivo*.***A*, representative illustration of HDGF levels by IHC staining and ROS accumulation by DHE fluorescence staining in WT and HDGF KO mice. The scale bars represent 200 μm (IHC staining) and 10 μm (DHE staining). *B*, quantification of hepatic DHE intensity between WT and HDGF-KO mice was qualified from six mice (n = 6). *C*, experimental scheme for noninvasive induction of orthotopic hepatoma in rats. Novikoff hepatoma was induced in the rat liver by ultrasound (US)-guided implantation of N1-S1 cells (*lower panel*, *red arrow* indicates injection on day 0), which was monitored by serial US analysis (*middle panel*, the tumor areas on day 14 are depicted by *dotted lines*) and verified after sacrifice on day 18. *D*, IHC analysis of HDGF expression in nontumor (N) and tumor (T) tissues from Novikoff hepatoma rat models. The scale bars represent 100 μm (*upper panel*) and 10 μm (*lower panel*). Further detection of ROS expression levels within nontumor and tumor tissues was achieved by using a DHE staining assay. The scale bar represents 10 μm. *E*, quantification of DHE signal intensity was achieved by using Image Pro-plus analysis software. Data were calculated from eight rats (n = 8). Here, DHE intensity data was caught from six random fields in each hepatic tissue and these data were further averaged. Finally, the acquired six or eight average values from mice (n = 6) and SD rats (n = 8) respectively were subjected to quantification. All data are expressed as the mean ± SD. ∗∗*p* < 0.01. DHE, dihydroethidium; HDGF, hepatoma-derived growth factor; IHC, immunohistochemistry; ROS, reactive oxygen species.

Since HDGF and oxidative stress are involved in liver carcinogenesis (9), it was hypothesized that HDGF might enhance ROS generation, thereby promoting HCC progression. By using *in situ* superoxide detection staining and HDGF KO mice, it was observed that the ROS-induced fluorescence intensity was significantly decreased in the livers of HDGF-KO mice compared with WT mice (Fig. 1, *A* and *B*). This finding suggests that genetic ablation of HDGF is associated with a reduction in endogenous ROS levels in the liver, which seemed to be consistent with our hypothesis. Subsequently, we examined the relationship between HDGF and ROS generation using the Novikoff hepatoma model induced by ultrasound-guided implantation (23). The advantage of this HCC model is that orthotopic HCC is created in immunocompetent animals within 14 days by noninvasive implantation of syngeneic hepatoma cells. Following animal sacrifice on day 18, orthotopic HCC was observed (Fig. 1*C*) and subjected to subsequent experiments. Immunohistochemical analysis and dihydroethidium (DHE) staining showed that HDGF expression and DHE fluorescence staining were significantly higher in tumor regions than in adjacent nontumor regions (Fig. 1, *D* and *E*). These results strongly support the notion that cellular HDGF levels are associated with endogenous ROS homeostasis *in vivo*.

### HDGF stimulates the ROS generation in hepatoma cells through NCL

**Figure 2:**
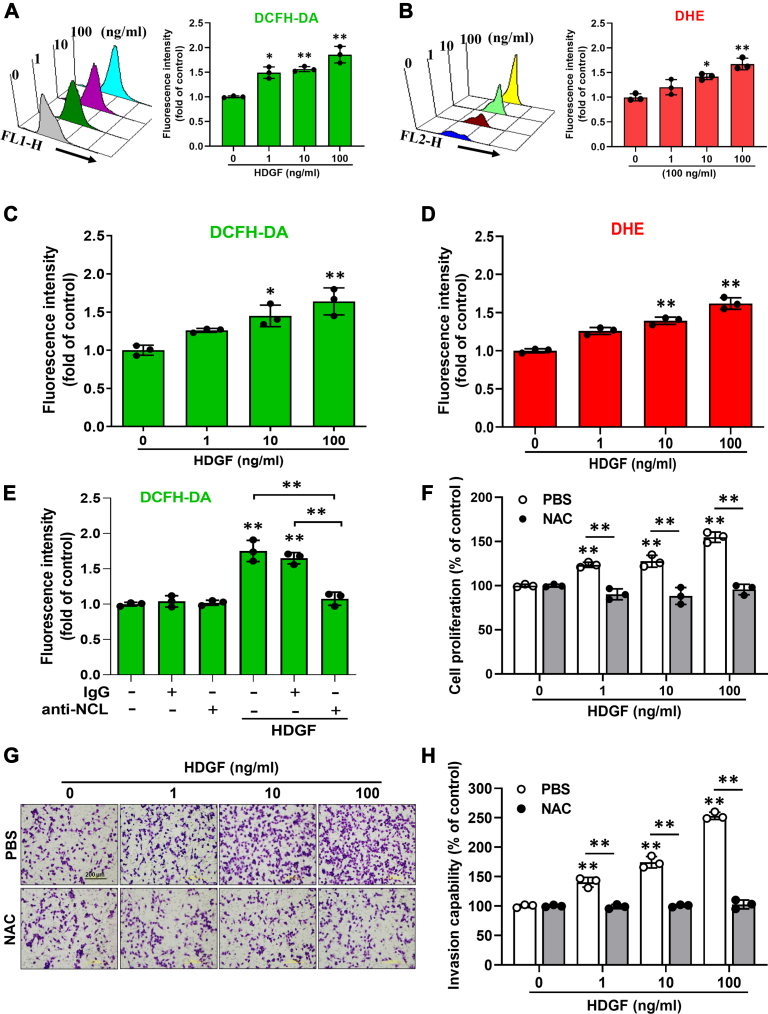
**Effect of recombinant HDGF administration on cellular ROS generation in human hepatoma cell lines**. Cells were treated with various doses of rHDGF for 4 h and then subjected to FACS analysis to determine ROS production using (*A*) DCFH-DA staining and (*B*) DHE staining. Moreover, similar results also found in Huh7 cells stained with (*C*) DCFH-DA staining and (*D*) DHE staining using FACS analysis. *E*, cells were incubated with anti-NCL antibodies (10 μg/ml) and rHDGF (10 ng/ml) for 4 h and immediately subjected to ROS analysis using DCFH-DA staining. *F*, moreover, SK-Hep-1 cells were pretreated with the antioxidant NAC (5 mM) for 1 h and then incubated with medium contained with 0.5% CS and various doses of rHDGF for 48 h. The cell growth rate was further detected and recorded using the MTT assay. *G*, representative pictures of invaded cells after rHDGF administration and incubation with or without NAC (5 mM). The scale bar represents 200 μm. *H*, the number of invaded cells was calculated from three different fields in each experimental group. All data are expressed as the mean ± SD of three experiments. ∗*p* < 0.05, ∗∗*p* < 0.01 compared with the indicated groups. DHE, dihydroethidium; DCFH-DA, 2′,7′-dichlorofluorescin diacetate; FACS, fluorescence activated cell sorting; HDGF, hepatoma-derived growth factor; MTT, 3-(4,5-dimethylthiazol-2-yl)-2,5-diphenyltetrazolium bromide; NAC, N-acetyl cysteine; NCL, nucleolin; rHDGF, recombinant HDGF; ROS, reactive oxygen species.

**Figure 3:**
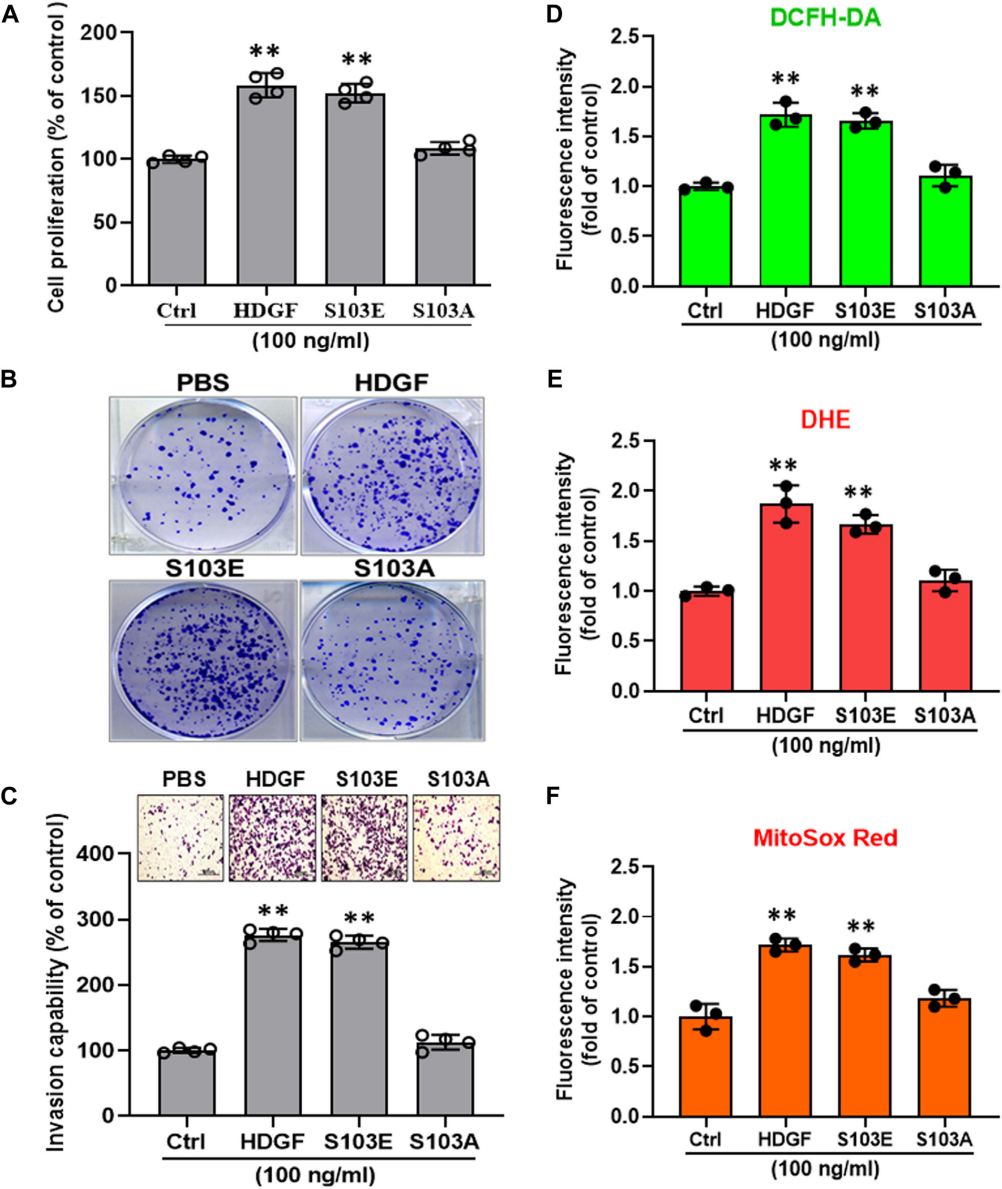
**Effects of S103E and S103A rHDGF mutants on cell proliferation, cell invasion, and ROS production in SK-Hep-1 hepatoma cells**. *A*, cells (5000 cells/well) were cultured in a 96-well plate and incubated with medium contained with 0.5% CS and rHDGF or rHDGF mutant proteins for 48 h. The effect of rHDGF mutant proteins on cell proliferation was determined using the MTT assay. Moreover, (*B*) the effect of rHDGF mutant proteins on colony formation was validated. Cells (2000 cells/well) were seeded in 6-well culture plates overnight and then cultured in medium contained with 0.5% CS and rHDGF or rHDGF mutant proteins for 7 to 10 days. The effect of exogenous HDGF and HDGF mutants on colony formation was further imaged and recorded using a photographic image assay. In addition, (*C*) to verify the effect of S103E and S103A rHDGF proteins on cell invasion, cells were incubated with rHDGF or rHDGF mutant proteins for 8 h, and then cell invasion was detected using a Boyden chamber assay. Quantification of invasive cells was performed in high-power fields. The scale bar represents 200 μm. Finally, the effects of the S103E and S103A rHDGF proteins on ROS generation were further analyzed. After cells were treated with rHDGF or rHDGF mutant proteins for 4 h and then subjected to staining with (*D*) DCFH-DA, (*E*) DHE, and (*F*) MitoSOX Red, and ROS production was analyzed using flow cytometry. The bar chart shows the quantification of fluorescence intensity. All data are expressed as the mean ± SD of three experiments. ∗*p* < 0.05; ∗∗*p* < 0.01 *versus* control group. CS, calf serum; DCFH-DA, 2′,7′-dichlorofluorescin diacetate; DHE, dihydroethidium; HDGF, hepatoma-derived growth factor; MTT, 3-(4,5-dimethylthiazol-2-yl)-2,5-diphenyltetrazolium bromide; rHDGF, recombinant HDGF; ROS, reactive oxygen species.

**Figure S1:**
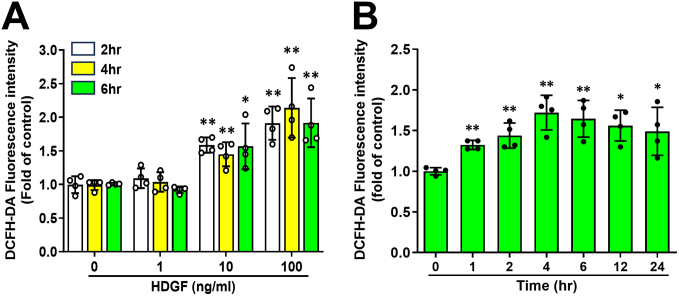
he time- and dose-effects of HDGF-mediated ROS generation in SK-Hep-1 cells using luminometer assay. *A*, after cells (3 × 10^5^ cells) were treated with various doses of HDGF (1, 10, 100 ng/ml) for short term of time course (2, 4, and 6 h), and Subsequently, cells were harvested and incubated with serum-free DMEM medium containing DCFH-DA (10 μM) for 20 min at 37 °C. One hundred microliters (∼2 × 10^4^ cells/well) of the cell suspension were dispensed into each well of a 96-well black view plate. Fluorescence intensity was quantified using a Polarstar luminometer (BMG Labtechnologies). *B*, time-dependent effect of HDGF on ROS production within 24 h. Cells were treated with HDGF (10 ng/ml) for the indicate time courses, ROS production were detected using a Polarstar luminometer. All data were mean ± SD of quadruplicates experiments.

**Figure S2:**
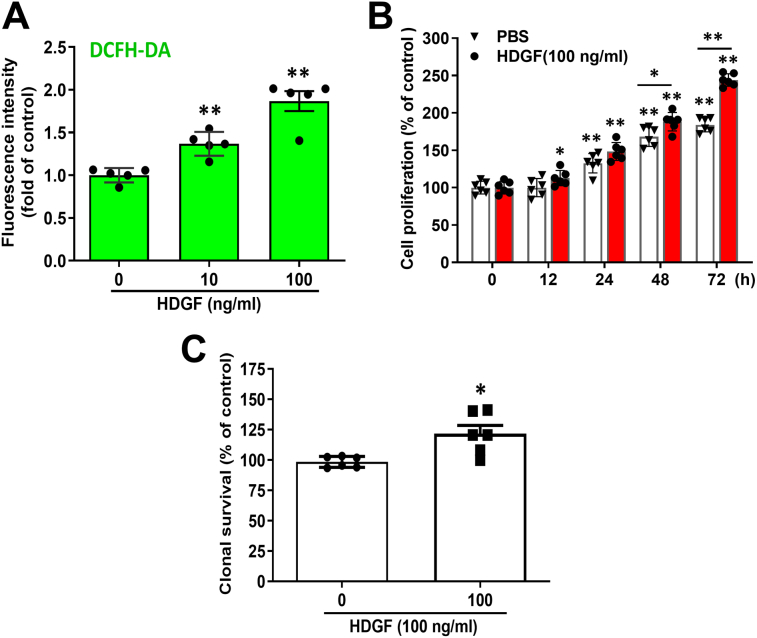
**Effects of HDGF on ROS induction, cell proliferation and clonal survival in non-transform cell mouse fibroblast NIH/3T3 cells.***A*, NIH/3T3 cells (1×10^6^ cells/dish in a 6-cm culture dish) were subjected to low serum (0.5%) starvation overnight, and then treated with HDGF (10 and/or 100 ng/ml) for 4 h. Cells were next harvested and stained with DCFH-DA (10 μg/ml) for 20 min at 37 °C. One hundred microliters (1 × 10^5^ cells per well) of the cell suspension were dispensed into each well of a 96-well black view plate. ROS Fluorescence intensity were determined using a Polarstar microplate reader. *B*, cells (5000 cells/ well in 96-well culture plate) were subjected to low serum (0.5%) starvation overnight, and then treated with HDGF (100 ng/ml) for various time courses (12, 24, 48, and 72 h). The cell proliferation was detected using an MTT assay. *C*, for clonal survival assay, cells (3000 cells/well in a 6-well culture plate) were cultured in a medium contained with 0.5% serum and HDGF (100 ng/ml) for 7-10 days. The clonal cells were next subjected to crystal violet staining assay and the cell clonal survival was determined using a micro-plate reader (BioTek® EON). The spectrometric absorbance at 595 nm was measured. All data are expressed as mean ± SD. ∗∗*p* < 0.05, ∗∗*p* < 0.01.

**Figure S3:**
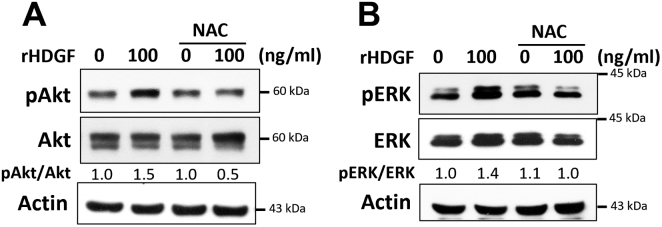
**Effect of NAC on HDGF-mediated Akt and ERK signaling in SK-Hep-1 cells.** Cells (3 × 10^5^ cells/well) were seeded in a 6-well culture plate. Cells were then pretreated with or without NAC (5 mM) in serum-free medium for 1 h, and then administrated with HDGF (100 ng/ml) for an additional 4 h. Cells were then harvested and subjected to analyze the protein activity of both (*A*) Akt and (*B*) ERK using Western blot assay.

Subsequently, we investigated whether exogenous HDGF enhanced ROS production in hepatoma cells. First, we validated the time- and dose-effects of HDGF-mediated ROS generation in SK-Hep-1 cells using 2′,7′-dichlorofluorescin diacetate (DCFH-DA) staining and luminometer assay. As shown in Fig. S1, a lower dose of HDGF (10 ng/ml) significantly elicits ROS generation within 2 h (Fig. S1*A*), and ROS level can be lasted at least for 24 h (Fig. S1*B*). This result suggests that HDGF significantly induces ROS production and maintains a higher intracellular ROS level for a while which would induce intracellular ROS accumulation and may offer conditions for subsequent liver tumorigenicity. Next, flow cytometry using DCFH-DA staining showed that the recombinant HDGF (rHDGF) protein dose dependently increased the intracellular ROS level by up to 2-fold in hepatoma cells (Fig. 2*A*). Consistently, DHE staining showed that rHDGF treatment significantly elevated intracellular ROS in a dose-dependent manner in SK-Hep-1 hepatoma cells (Fig. 2*B*). Similar results were also observed in Huh7 cell lines (Fig. 2, *C* and *D*). On the other hand, to demonstrate the effects of HDGF-mediated ROS generation on contributing oncogenesis not only in tumor cells but also in nontumor cells, we thereby validate the effects of HDGF on ROS induction, cell proliferation, and clonal survival in the nontransform cell-mouse fibroblast NIH/3T3 cells. As shown in Fig. S2, our data indicated that HDGF significantly induces ROS production in a dose-dependent manner (Fig. S2*A*) and promotes cell proliferation in a time-dependent manner (Fig. S2*B*) as well as elicits clonal survival (Fig. S2*C*). Our data are consistence with previous studies which demonstrated that HDGF significantly induces cell proliferation in NIH/3T3 cells and elicits the generation of sarcomatous tumors in nude mice through the induction of VEGF (21). Altogether, these results indicate that HDGF potent induces ROS generation may involve in tumorigenicity in not only tumor cells but also nontransform cells. In addition, HDGF is a mitogen and phosphoprotein. Phosphorylation of S103 in HDGF has been demonstrated that involved in mitogenic activity, cell cycle progression, and cell proliferation (24). Here, to further verify the role of HDGF-mediated ROS generation contributed to oncogenic behaviors, we generated two HDGF mutant proteins including Ser103Ala (S103A) and Ser103Glu (S103E) and analyzed their effects on oncogenic behaviors and ROS generation. As shown in Fig. 3, S103A rHDGF mutant significantly reversed the HDGF-mediated cell proliferation (Fig. 3*A*), colony formation (Fig. 3*B*), and cell invasion capability (Fig. 3*C*). By further detecting the effects of rHDGF mutants on ROS generation, we found that the S103A mutant but not the S103E mutant obviously abolished HDGF-stimulated ROS production not only in intracellular ROS (Fig. 3, *D* and *E*) but also in mitochondrial ROS (Fig. 3*F*). Whereas an S103E phospho-mimic mutation was constitutively active, resulting in an increased survival activity and ROS generation relative to the S103A rHDGF mutant. These results suggest that exogenous HDGF may induce ROS accumulation in hepatoma cells. Since it has been reported that surface NCL transmits the oncogenic signaling of HDGF (22), we evaluated whether blocking NCL signaling affected HDGF-induced ROS generation in hepatoma cells. Antibody neutralization of NCL effectively abolished the HDGF-induced increase in hydrogen peroxide levels in hepatoma cells. (Fig. 2*E*). Therefore, HDGF promotes ROS generation in hepatoma cells *via* NCL. To validate the role of ROS generation in HDGF-induced hepatocarcinogenesis, we evaluated the influence of the antioxidant N-acetyl cysteine (NAC) on HDGF-stimulated oncogenic behaviors in hepatoma cells. Proliferation assays showed that NAC dramatically attenuated HDGF-stimulated proliferation in hepatoma cells (Fig. 2*F*). Likewise, NAC treatment also perturbed the HDGF-induced invasiveness of hepatoma cells (Fig. 2, *G* and *H*). Indeed, the effects of NAC on HDGF-mediated cell proliferation- and invasion-associated signaling including Akt and MAP signaling such as ERK activity were further validated. As shown in Fig. S3, our data revealed that HDGF significantly induced cell proliferation-associated signaling activation such as Akt signaling and ERK signaling. Moreover, NAC treatment also obviously abolished HDGF-mediated both Akt signaling and ERK signaling. This result suggests that HDGF-elicited ROS generation is involved in cell proliferation and invasion through the upregulation of Akt signaling and MAPK kinase-mediated signaling. In summary, these findings suggest that activation of HDGF/NCL signaling triggers an increase in intracellular ROS, thereby contributing to hepatoma progression.

### HDGF regulates ROS generation in mitochondria of hepatoma cells

**Figure 4:**
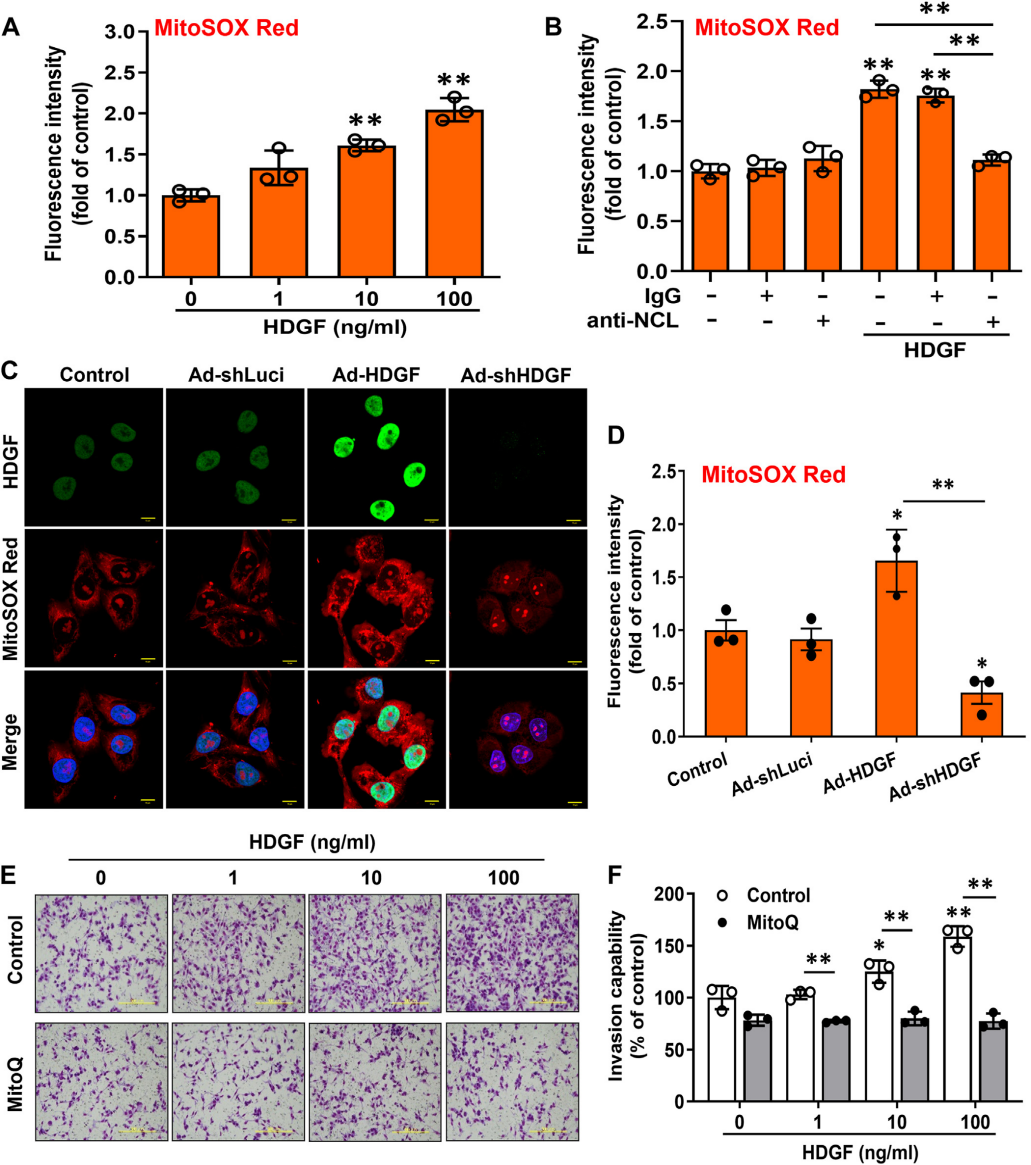
**Effect of HDGF on mitochondrial ROS generation in SK-Hep-1 cells.***A*, cells were treated with various doses of rHDGF for 4 h, and HDGF-mediated mitochondrial ROS generation was detected using MitoSOX Red staining and flow cytometry. Data are expressed as the fold change compared with the control group. *B*, the effect of NCL antibody neutralization on HDGF-induced mitochondrial ROS generation. *C*, representative pictures showing changes in HDGF expression by the adenoviral gene delivery system and the effect on mitochondrial ROS production, as detected by MitoSOX Red staining and fluorescence microscopy. The scale bar represents 10 μm. *D*, quantification of the MitoSOX Red fluorescence intensity by Image Pro-plus analysis software. *E*, representative pictures of invaded cells after HDGF administration and incubation with or without Mito Q. The scale bar represents 200 μm. *F*, the number of invaded cells was calculated from three different fields for each experimental group. All data are expressed as the mean ± SD of three experiments. ∗*p* < 0.05, ∗∗*p* < 0.01 compared with the indicated groups. HDGF, hepatoma-derived growth factor; NCL, nucleolin; rHDGF, recombinant HDGF; ROS, reactive oxygen species.

Mitochondria are the major source of intracellular ROS production in mammalian cells. Thus, we elucidated the role of mitochondria in HDGF-induced ROS production. Flow cytometry and the mitochondria-specific MitoSOX Red dye showed that HDGF treatment significantly increased the fluorescence intensity of MitoSOX Red in hepatoma cells in a dose-dependent manner (Fig. 4*A*). In addition, NCL neutralization abrogated HDGF-induced mitochondrial ROS production in hepatoma cells (Fig. 4*B*). We used adenoviral gene delivery to modulate cellular HDGF levels, and HDGF overexpression prominently augmented MitoSOX Red staining in hepatoma cells (Fig. 4, *C* and *D*). In contrast, silencing HDGF correlated with a decrease in MitoSOX Red fluorescence intensity. Finally, we used the specific mitochondria-targeted antioxidant MitoQ to disrupt the stimulatory effect of HDGF on the invasiveness of hepatoma cells (Fig. 4, *E* and *F*). Overall, mitochondria seem to play a critical role in mediating HDGF-induced ROS generation and liver carcinogenesis.

### HDGF promotes the oxygen consumption, extracellular acidification, and oxidative phosphorylation in hepatoma cells

**Figure 5:**
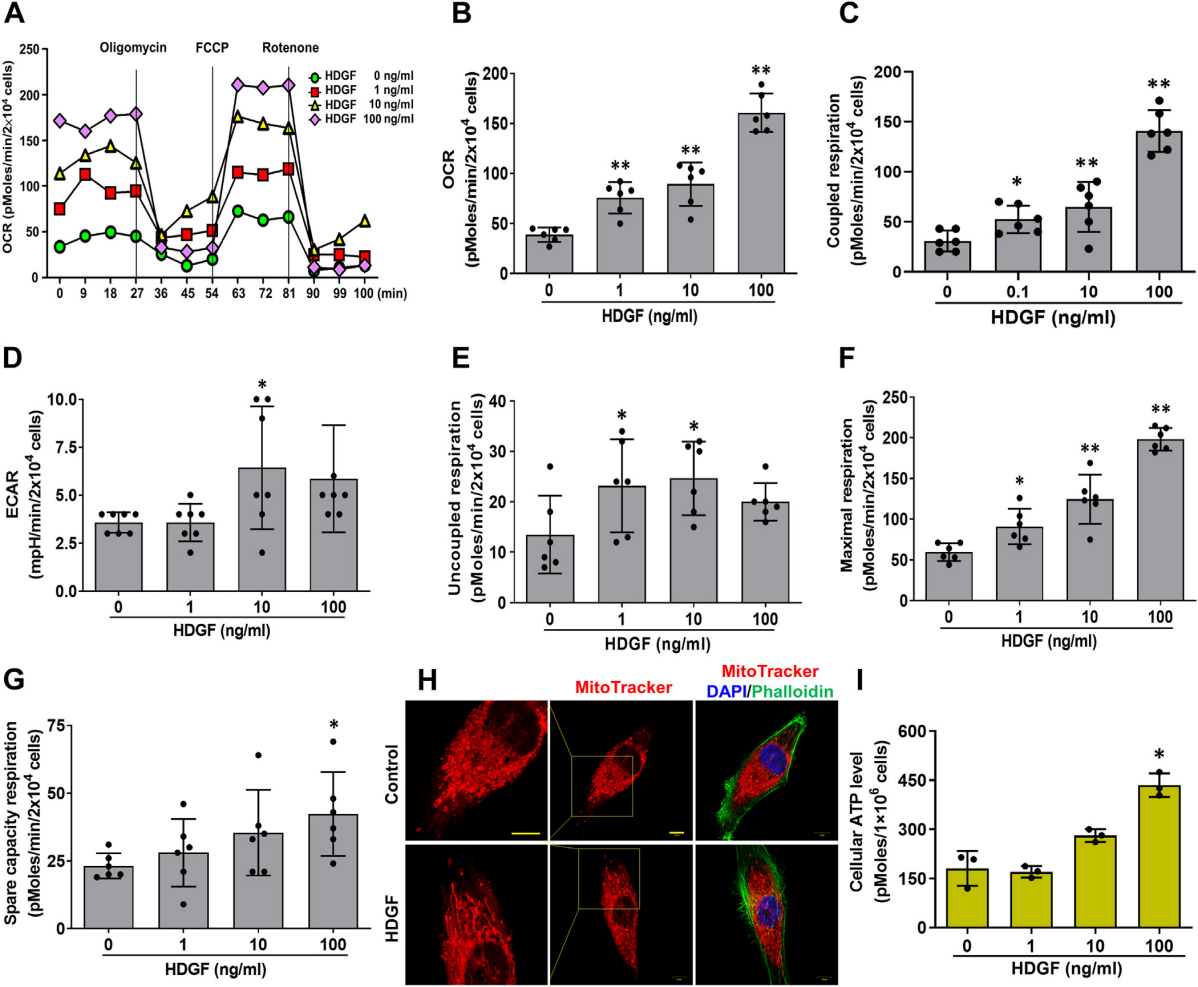
**Effect of exogenous HDGF on mitochondrial bioenergetics in hepatoma cells.***A*, OCR in response to HDGF protein treatment was detected by the Seahorse XF24 analyzer in the absence or presence of oligomycin (1 μM), FCCP (0.5 μM), and rotenone (1 μM) by counting 5 × 10^4^ cells. Comparison of mitochondrial bioenergetic parameters, including (*B*) OCR, (*C*) coupled respiration, (*D*) ECAR, (*E*) uncoupled respiration, (*F*) maximal respiration, and (*G*) spare respiration, in cells treated with rHDGF. *H*, HDGF-induced mitochondrial morphological alterations were monitored by tracing three selected probe signals that recognized three different cellular organelles: MitoTracker (*red*) for mitochondria, phalloidin (*green*) for F-actin, and DAPI for nuclei (*blue*). The scale bars represent 5 μm (*left panel*) and 10 μm (*middle* and *right panels*). *I*, total cellular ATP levels were measured using a luciferase-based luminescence assay kit. All data were calculated from three independent experiments. ∗*p* < 0.05, ∗∗*p* < 0.01. DAPI, 4′,6-diamidino-2-phenylindole; ECAR, extracellular acidification rate; FCCP, carbonyl cyanide-p-trifluoromethoxyphenylhydrazone; HDGF, hepatoma-derived growth factor; OCR, oxygen consumption rate; rHDGF, recombinant HDGF.

**Figure S4:**
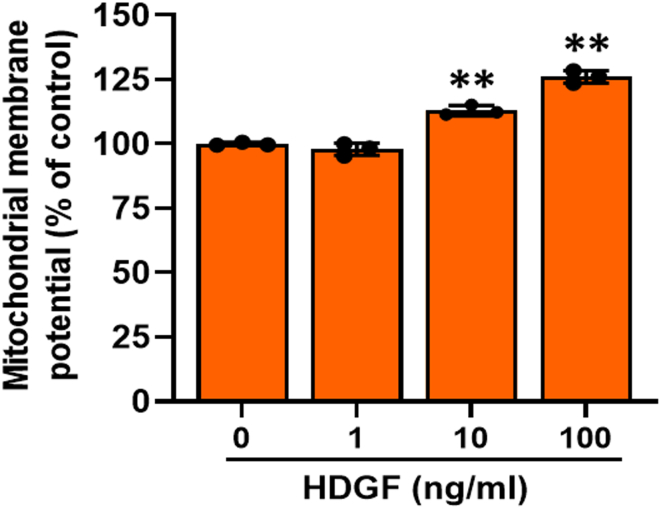
**Effect of HDGF on the mitochondrial membrane potential of SK-Hep-1 cells.** Cells (3 × 10^5^ cells/well) were seeded in a 6-well plate and serum starvation overnight. Cells were next treated with various doses of rHDGF (0, 1, 10, 100 ng/ml) for 4 h. Cells were then incubated with TMRM (100 nM, Sigma-Aldrich) for 20 min at 37 °C in dark and next subjected to FACS analysis. Data are expressed as the mean ± SD. ∗∗*p* < 0.01 *versus* control group.

**Figure S5:**
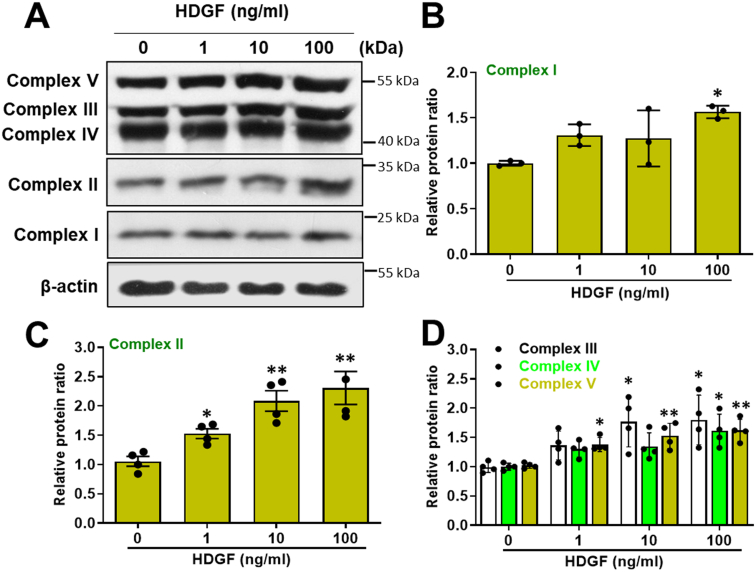
The effect of exogenous HDGF on mitochondrial respiration chain complexes in hepatoma SK-Hep-1 cells. Cells were treated with HDGF (1, 10, 100 ng/ml) for 4 h. *A*, HDGF increased the protein levels of mitochondrial complexes I – V in a dose-dependent manner. *B*–*D*, five complexes were further quantified respectively and expressed as mean fold change ± SD. ∗*p* < 0.05; ∗∗*p* < 0.01 *versus* control group.

Mitochondrial bioenergetics, including oxidative phosphorylation (OXPHOS), ATP biosynthesis and mitochondrial dynamics, are highly relevant to liver tumorigenesis (8, 25, 26). We used Seahorse metabolic flux assays to characterize the influence of HDGF on mitochondrial bioenergetics in SK-Hep-1 hepatoma cells. As shown in Figure 5*A*, various inhibitors were used to analyze the time-dependent profile of the oxygen consumption rate (OCR) in HDGF-treated SK-Hep-1 cells. Our data revealed that HDGF significantly increased the basal OCR and coupled respiration in a dose-dependent manner (Fig. 5, *B* and *C*). This finding indicated that HDGF enhanced mitochondrial oxidative phosphorylation through a series of substrate oxidations, an electrochemical proton gradient between the mitochondrial intermembrane space and matrix, and ATP synthesis and increased the mitochondrial basal anabolic rate and ATP production (27). Moreover, the profile of the extracellular acidification rate (ECAR) showed the HDGF dose dependently mediated aerobic glycolysis for energy consumption (Fig. 5*D*). Next, we demonstrated that HDGF significantly increased nonelectron transporter chain respiration (uncoupled respiration) (Fig. 5*E*), maximal oxygen consumption (Fig. 5*F*), and spare capacity respiration (Fig. 5*G*). In addition, staining with the mitochondria-selective dye MitoTracker showed that the HDGF-induced significant fusion and tubular formation in mitochondria rather than the fragmented morphology observed in the control group (Fig. 5*H*). Further ATP assays suggested that HDGF significantly enhanced cellular ATP production in a dose-dependent manner (Fig. 5*I*). In addition, we analyzed the effects of HDGF on the mitochondrial membrane potential using a tetramethylrhodamine, methyl ester labeling dye and fluorescence-activated cell sorting (FACS) analysis also demonstrated that HDGF significantly elicited the upregulation of mitochondrial membrane potential in a dose-dependent manner (Fig. S4). Moreover, HDGF treatment also triggers the upregulation of mitochondrial respiratory chain complexes in a dose-dependent manner (Fig. S5). Therefore, these results implied that HDGF not only elevates mitochondrial bioenergetics but also promotes oxidative phosphorylation and mitochondrial dynamics which may explain why both increased OCR and ROS levels in HDGF treatment. Indeed, accumulating evidence has suggested that mitochondria can be reprogrammed in proliferating cancers to guarantee the high energy requirements for cell division, migration, and invasion (28). For example, some tumors are highly dependent on OXPHOS for ATP through genetic alterations such as the SMARCA4 mutant lung cancer (29); and the highly bioenergetic reliance on mitochondrial fatty acid oxidation to support tumor growth is found in MYC-overexpressing triple-negative breast cancer (30); as well as high expression of mitochondrial respiratory complex I components is found in pancreatic cancer which has identified as a high OXPHOS tumor (31). Accordingly, developing molecular therapeutics targeting OXPHOS and the ETC in cancer has become an emerging target in cancer therapy (32). Altogether, these results suggest that HDGF-mediated elevating mitochondrial bioenergetics and promoting oxidative phosphorylation play important roles in liver carcinogenesis.

### Antibody neutralization of surface NCL abrogates the HDGF-induced oxygen consumption and extracellular acidification in hepatoma cells

**Figure 6:**
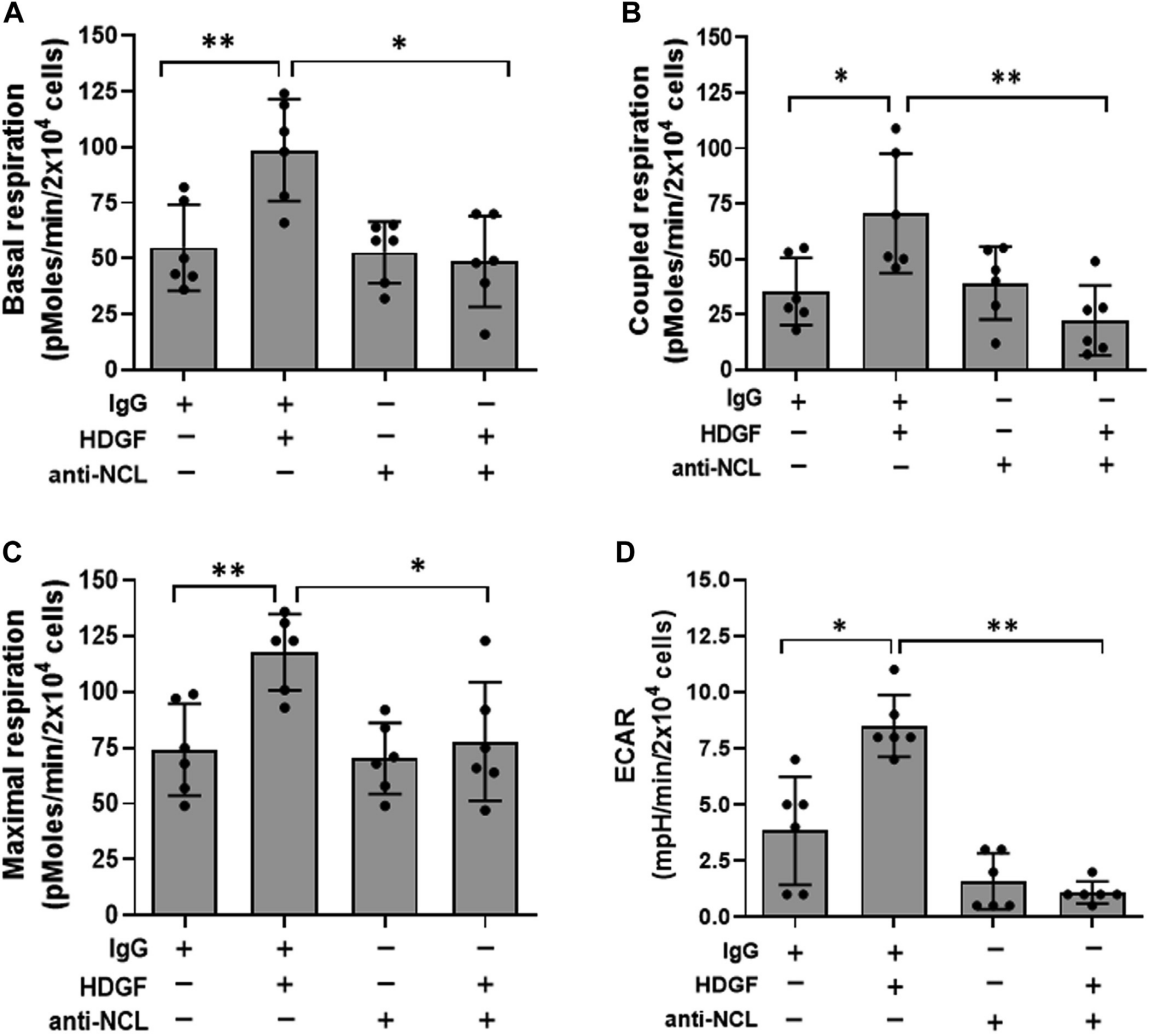
**Effects of NCL neutralization on HDGF-induced bioenergetics in hepatoma cells**. SK-Hep-1 hepatoma cells were incubated with anti-NCL antibodies (10 μg/ml) and rHDGF (10 ng/ml) for 4 h before mitochondrial bioenergetics analysis. *A*, the basal respiration, (*B*) coupled respiration, (*C*) maximal respiration, and (*D*) ECAR were determined using the Seahorse XF24 analyzer. Data are the mean ± SD of three experiments. ∗*p* < 0.05, ∗∗*p* < 0.01. ECAR, extracellular acidification rate; HDGF, hepatoma-derived growth factor; NCL, nucleolin; rHDGF, recombinant HDGF.

**Figure S6:**
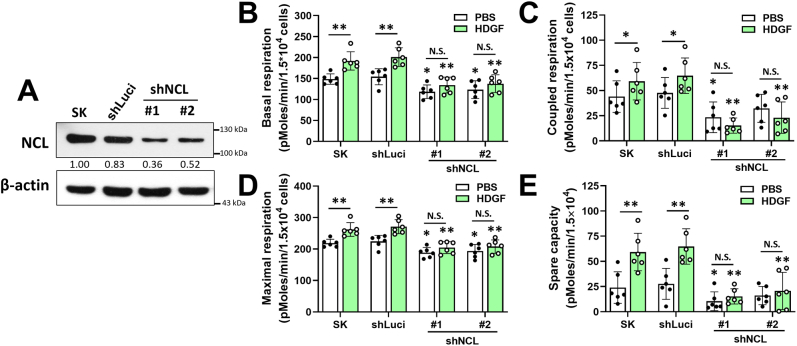
Effects of NCL knockdown on HDGF-induced bioenergetics in hepatoma cells. Two shNCL stable clones from SK-Hep-1 cells were generated as described in the “material and methods” section in the resubmitted manuscript. *A*, the NCL knockdown efficiency was evaluated using Western blotting. *B*, to validate the effect of HDGF on bioenergetics of NCL knockdown clones, cells (1.5 × 10^4^ cells/well) were seeded in 8-well culture plate and serum starvation for overnight. Cells were then treated with or without HDGF protein (100 ng/ml) for 4 h and then subjected to mitochondrial bioenergetics analysis. *A*, the basal respiration, (*B*) coupled respiration, (*C*) maximal respiration and (*D*) spare capacity were determined using the Seahorse XF HS Mini analyzer. Data are expressed as the mean ± SD. ∗*p* < 0.05; ∗∗*p* < 0.01 *versus* control groups. N.S., not significant.

Since we demonstrated that HDGF induces ROS generation in hepatoma through surface expression of NCL, it seems plausible that HDGF/NCL signaling might modulate the activity of mitochondrial bioenergetics to promote an increase in ROS in hepatoma cells. Seahorse metabolic flux assays showed that NCL neutralization significantly abolished the HDGF-induced increase in basal respiration (Fig. *A*), coupled respiration (Fig. 6*B*), and maximal oxygen consumption (Fig. 6*C*) in hepatoma cells. Interestingly, application of an anti-NCL antibody not only attenuated the basal ECAR but also completely abrogated the HDGF-stimulated ECAR in hepatoma cells (Fig. 6*D*). In addition, similar results are also found in NCL knockdown stable clones. Here, we generated two stable clones including shNCL#1 and shNCL#2 for analyzing the effects of NCL knockdown on HDGF-induced bioenergetics in SK-Hep-1 hepatoma cells. As shown in Fig. S6*A*, at least 50% of NCL knockdown was achieved in these shNCL clones as compared to either SK-Hep-1 or shLuci group by using Western blot assay. Next, we detected the effect of HDGF on mitochondrial bioenergetics in two shNCL#1 and shNCL#2 clones using the Seahorse XF HS Mini analyzer. As shown in Fig. S6, *B*–*E*, Seahorse metabolic flux assays showed that NCL knockdown obviously attenuated the mitochondrial bioenergetics. Moreover, NCL knockdown significantly abolished the HDGF-induced increase in basal respiration (Fig. S6*B*), coupled respiration (Fig. S6*C*), maximal oxygen consumption (Fig. S6*D*), and spare capacity (Fig. S6*E*) in hepatoma cells. These data suggest that HDGF regulates mitochondrial bioenergetics in hepatoma cells through the NCL axis.

### Genetic silencing of SOD2 augmented the HDGF-induced tumorigenicity and ROS accumulation in hepatoma cells

**Figure 7:**
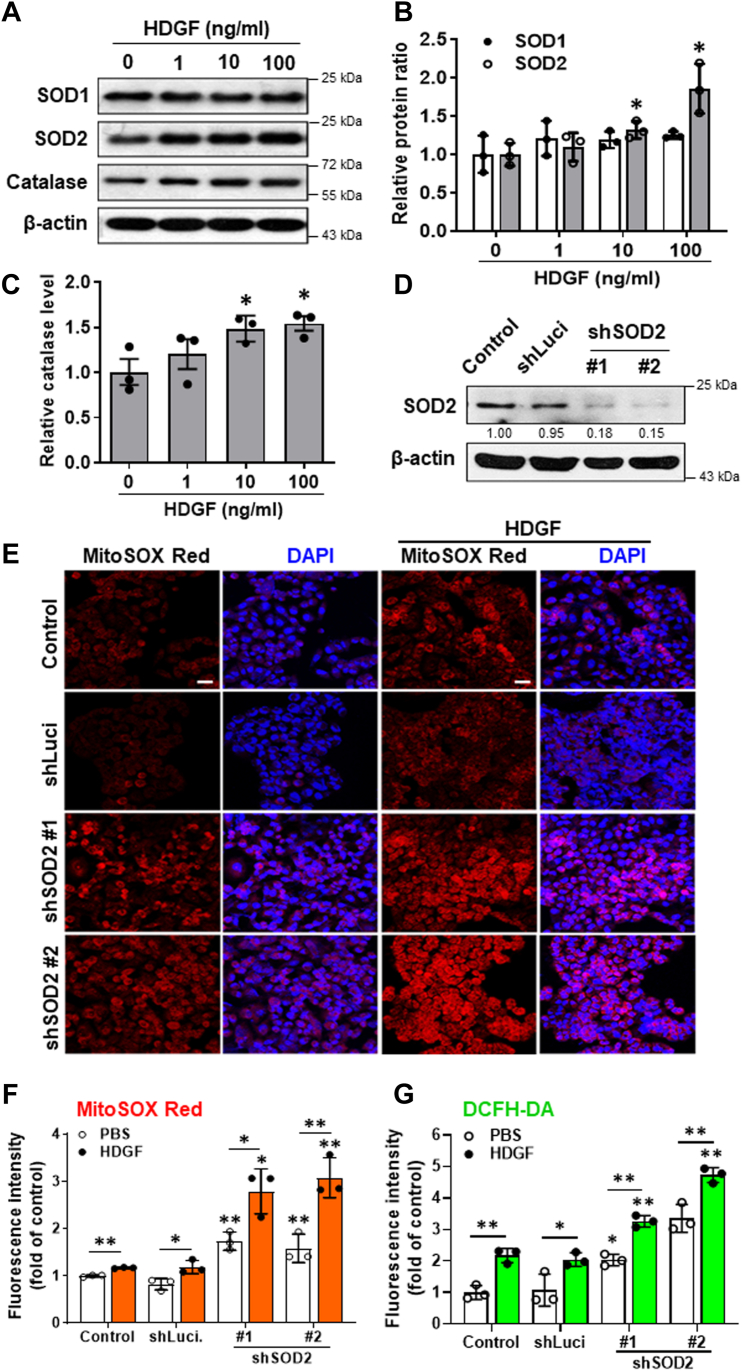
**Effect of SOD2 silencing on HDGF-induced mitochondrial ROS production in SK-Hep-1 cells**. *A*, the protein levels of SOD1, SOD2 and catalase were detected by Western blotting. *B* and *C*, the relative protein ratio was normalized to β-actin using Image Pro-plus analysis software. Data are expressed as the fold change ± SEM from duplicate experiments. *D*, Western blot analysis of the basal level of SOD2 in SOD2 knockdown stable clones. *E*, illustration of mitochondrial ROS levels in SOD2 knockdown stable clones incubated with or without HDGF using MitoSOX Red staining and fluorescence imaging. The scale bar represents 50 μm. *F*, the intensity of MitoSOX Red fluorescence was quantified using Image Pro-plus analysis software. *G*, the effects of HDGF (10 ng/ml) on the subcellular ROS generation of these SOD2 knockdown stable clones using DCFH-DA staining and FACS analysis. Data are expressed as the mean ± SD of three experiments. ∗*p* < 0.05, ∗∗*p* < 0.01 *versus* the control group. DCFH-DA, 2′,7′-dichlorofluorescin diacetate; FACS, fluorescence activated cell sorting; HDGF, hepatoma-derived growth factor; ROS, reactive oxygen species; SOD2, superoxide dismutase 2.

Oncogenic cytokines such as TGF-β enhance ROS accumulation *via* the downregulation of free radical scavenging enzymes. Hence, we elucidated the influence of HDGF on the expression of these ROS-clearing enzymes in hepatoma. Immunoblot analysis showed that rHDGF treatment induced SOD2 upregulation in a dose-dependent manner but did not affect SOD1 protein levels in hepatoma cells (Fig. 7, *A* and *B*). In addition, HDGF significantly increased catalase protein levels in hepatoma cells (Fig. 7*C*). Thus, unlike TGF-β, HDGF upregulated rather than downregulated the expression of free radical scavenging enzymes in hepatoma cells. It seems plausible that an increase in SOD2 and catalase expression was induced to counteract the HDGF-induced ROS elevation. An alternative explanation of the data is that HDGF plays a role in maintaining tumor cell activity. Because cell proliferation and invasion require energy, HDGF may activate respiration for energetic support. Because a smaller increase of ROS is possible with increased OCR, this small ROS increase may act as a signaling molecule for proliferation and invasion processes.

SOD2 is critical for the clearance of superoxide anions in mitochondria (33). To investigate the role of SOD2 in HDGF-mediated mitochondrial ROS generation, SOD2-silenced stable clones were generated using lentiviral short hairpin RNA-mediated knockdown and verified by immunoblot analysis (Fig. 7*D*). Fluorescence microscopy and MitoSOX Red staining showed that mitochondrial ROS generation was prominently elevated in SOD2-silenced hepatoma cells. Furthermore, HDGF-induced ROS production in mitochondria was more pronounced in these SOD2 knockdown stable clones. We next detected the effect of HDGF on mitochondrial ROS generation in two SOD2 knockdown stable clones. MitoSOX Red staining showed that HDGF significantly aggravated mitochondrial ROS production in SOD2 knockdown stable clones (Fig. 7, *E* and *F*). Consistently, these HDGF-depleted hepatoma cells had significantly higher invasiveness and ROS levels, as shown by DCFH-DA staining and FACS assay (Fig. 7*G*). Therefore, these results suggest that cells upregulated SOD2 and catalase in response to HDGF-accelerated mitochondrial bioenergetics and mitochondrial ROS generation.

## Discussion

**Figure 8:**
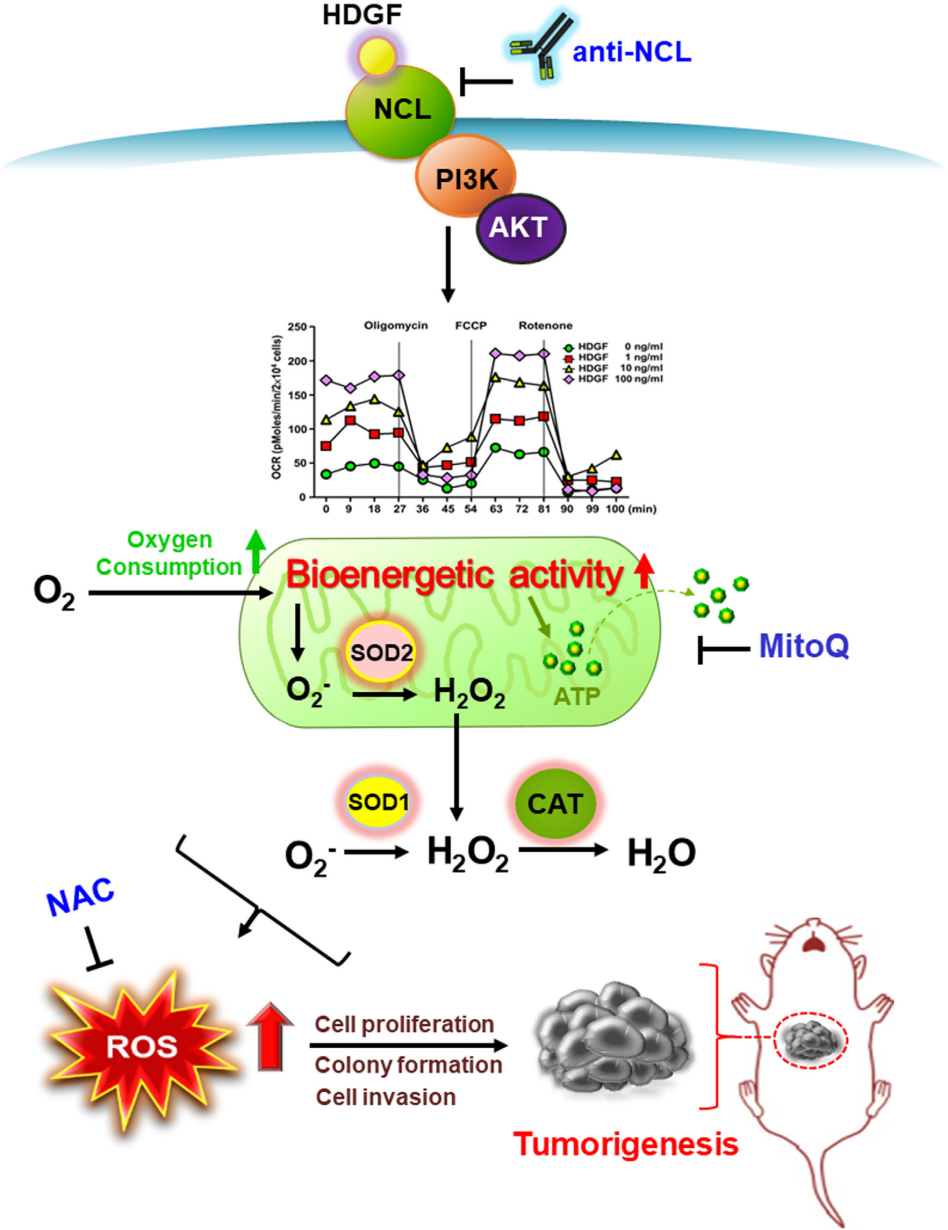
**Schematic diagram illustrating the role of HDGF-induced ROS generation in promoting liver carcinogenesis**. HDGF interacts with surface NCL and triggers activation of the PI3K/Akt pathway, which promotes mitochondria bioenergetics to enhance ROS and ATP production in mitochondria in hepatoma cells. An exogenous supply of antioxidants such as glutathione precursor NAC or mitochondria-targeted antioxidant-MitoQ inhibits the HDGF-induced proliferation and invasion, indicating ROS generation plays a pivotal role in HDGF-mediated tumorigenesis. Finally, genetic knockdown or antibody neutralization of NCL abolishes the HDGF-mediated mitochondrial bioenergetics and ROS generation in mitochondria, suggesting that the HDGF/NCL signaling axis stimulates oxygen consumption and mitochondrial ROS generation, ultimately contributing to liver tumorigenesis. HDGF, hepatoma-derived growth factor; NAC, N-acetyl cysteine; NCL, nucleolin; ROS, reactive oxygen species.

The present study first revealed that ROS accumulation in HCC tissues was positively correlated with HDGF expression. We subsequently demonstrated that adding rHDGF protein strikingly escalated ROS generation in both Huh7 and SK-Hep-1 cells, whereas abolishing ROS production by NAC and MitoQ was sufficient to abrogate the stimulatory effect of HDGF on cell viability and invasion. Moreover, we further determined that ROS generation by HDGF increased mitochondrial bioenergetic activity. Finally, interruption of the HDGF/NCL axis by antibody neutralization was able to eliminate the accumulation of ROS and mitochondrial bioenergetic activity. Therefore, we propose a schematic model for the activation of the HDGF/NCL signaling axis, which stimulates oxygen consumption and mitochondrial ROS generation and ultimately contributes to liver tumorigenesis (Fig. 8).

Cellular ROS can be produced from the mitochondrial ETC, endoplasmic reticulum system, xanthine oxidase, cyclooxygenase, and NOXs (34). Several stimuli can induce cellular ROS generation, such as growth factors, enzymes, cytokines, and protein kinases (13). TGF-β induces and interplays with ROS and plays an important role in cancer progression, which involves Smad and non-Smad pathways and active receptor complex-mediated PI3K/Akt/Rho GTPase signaling, as well as NOX signaling activation (5, 35). Moreover, TNF induces ROS-mediated signaling through the activation of NOX1 and NOX2 complexes and thus regulates numerous biological processes, including cell survival, apoptosis, and metabolism. Dysregulation of TNF-mediated ROS signaling is a characteristic of inflammatory diseases and cancers (36).

In our previous study, we demonstrated that the HDGF expression level was highly correlated with human HCC progression (14). In this study, we further verified that HDGF-mediated cellular ROS generation plays pivotal roles in liver tumorigenesis in both a rat orthotopic HCC model and HDGF KO mouse models. Moreover, we showed that rHDGF or modulating HDGF by an adenovirus delivery system could accelerate cellular ROS generation, as shown by 2′, 7′ –dichlorofluorescein and MitoSOX Red staining in hepatoma cells. Additionally, selective antioxidants, including diphenyleneiodonium (for NADPH oxidase), allopurinol (for xanthine oxidase), and indomethacin (for cyclooxygenases), did not abolish the stimulatory effect of HDGF on ROS production (data not shown). Therefore, our results suggest that HDGF-induced ROS generation primarily involves mitochondria.

Previous studies have indicated that the Nox family serves as a prominent source of ROS in HCV-infected hepatocytes (37, 38). HCV significantly upregulates the protein expression and activity of NOX1, NOX2, and NOX4, as well as TNFα, enhancing ROS production in hepatocytes. This orchestration among HCV, NOXs, and TNF thus promotes chronic hepatitis, cirrhosis, and HCC development (5). In addition, TGF-β-induced ROS production involved in NOX2 and NOX4 signaling activation also contributes to HCV-mediated liver fibrosis and HCC progression (5, 35, 38). As a biomarker of tumorigenesis, HDGF is not only an inducer of tumor progression but also an indicator of tissue inflammation. For instance, neutrophils stimulate an inflammatory TNF-α/HDGF/COX-2 signaling cascade that plays an important role during *Helicobacter pylori*-induced gastritis and gastric carcinogenesis (39). HDGF induces TNF-α/IL-1β/IL-6/COX-2 signaling in response to concanavalin A-induced hepatitis *in vitro* and *in vivo* (16). Moreover, HDGF can interact with ROS-mediated inducers and/or signaling to induce tumorigenic progression. HDGF also accelerates the formation of hepatic fibrosis by collaborating with TGF-β in murine models (15). Furthermore, HDGF binds to membrane NCL to activate the HIF-1α/VEGF axis, and Akt/NFκB signaling contributes to poor disease control in oral cancer (40). Other research groups have indicated that TGF-β stimulates HDGF expression *via* activation of the transcription factor HIF-1α in pancreatic stellate cells (41). Although the precise mechanisms of HDGF-mediated mitochondrial ROS generation in HCC are still unclear, the orchestration among HDGF with TGF-β, NOXs, and TNF to upregulate mitochondrial ROS-mediated signaling may play pivotal roles in liver tumorigenesis.

HDGF recruits NCL from the nucleus to the cell membrane and triggers downstream PI3K/AKT signaling activation, which ultimately results in liver carcinogenesis (22). The present study showed that blocking NCL with antibody neutralization was sufficient to abrogate the effects of HDGF-mediated ROS production and mitochondrial activities. Accumulating evidence has indicated that AKT is activated by PI3K-mediated signaling and can translocate to the mitochondrial matrix and inner membrane (42), and activated AKT may suppress mitochondrial glycogen synthase kinase 3 beta activity, alleviating the negative regulation of pyruvate dehydrogenase and α-ketoglutarate dehydrogenase complexes and resulting in enhanced mitochondrial bioenergetics and ROS generation (43, 44, 45). Moreover, PI3K/AKT signaling is involved in peroxisome proliferator-activated receptor-γ coactivator-1α-mediated mitochondrial bioenergetic activity (46). Therefore, our results suggest that HDGF-mediated promotion of mitochondrial bioenergetics occurs *via* the NCL-mediated PI3K/AKT signaling pathway.

Increased ROS within cells is associated with abnormal cancer cell growth and indicates an imbalance in redox homeostasis that can be attributed to increased ROS production or the dysregulation of ROS-scavenging capacity (47). Moreover, persistent intrinsic ROS accumulation can “train” cancer cells to develop a higher antioxidant capacity, which makes malignant cells resistant to adverse conditions and exogenous stress (48). Mitochondria are highly active organelles and govern cellular redox homeostasis by transferring and sponging more than 90% of electrons from the ETC to generate superoxide anion (O_2_^․–^) (49). Reactive O_2_^․–^ can be converted into H_2_O_2_ by SOD1, SOD2, and SOD3 in the cytosol, mitochondrial matrix, and extracellular space, respectively. H_2_O_2_ is further reduced into H_2_O by catalase and the peroxidase system, including the thioredoxin/thioredoxin reductase/peroxiredoxin (Trx/TrxR/Prx) and glutathione/glutathione peroxidase (GSH/GPx) systems (50). In this study, we studied the ROS scavenger enzyme expression profiles in response to the administration of rHDGF to hepatoma cells. Our data showed that HDGF promoted catalase and SOD2 expression but did not affect SOD1 levels in SK-Hep1 cells. SOD2 knockdown aggravated mitochondrial ROS accumulation in HDGF-treated cells, implying that the HDGF-mediated increase in mitochondrial ROS scavengers is a response to an increase in mitochondrial activity through the upregulation of mitochondrial levels and/or enhanced mitochondrial fusion activity, thereby causing ROS accumulation. Accordingly, our results suggest that HDGF-induced ROS generation occurs primarily by accelerating mitochondrial bioenergetics and further upregulates endogenous antioxidant enzymes, including SOD2 and catalase, to modulate ROS tolerance of mitochondria and maintain the proliferation and invasion of hepatoma cells.

Indeed, an increasing number of studies have demonstrated the multiple roles of SOD2 in cancer progression, metastasis, and tumor inhibition in different types of cancer and tumor stages. For instance, SOD2 regulates transcription factors such as NFκB, HIF-1, AP-1, and p53 in a redox-dependent manner to modulate cell proliferation, transformation, migration, invasion, and angiogenesis. Moreover, the upregulation of SOD2 contributes to anoikis resistance, prolonging tumor cell survival. Furthermore, SOD2 can play dual roles in tumor suppression or metastatic disease progression at initial stages and later stages of tumor progression, respectively (51). Previous studies indicated that altered antioxidant molecule expression levels significantly enhanced tumor development in animal models, such as mice that were deficient in SOD1 that developed nodular hyperplasia or HCC (52); transgenic mice overexpressing glutathione peroxidase and/or SOD1 had enhanced progression of skin carcinogenesis (53). Accordingly, further verifying the modulatory mechanism between SOD2 and HDGF in HCC progression is a critical issue. Moreover, other mitochondrial antioxidant peroxidase systems, such as the Trx/TrxR/Prx and GSH/GPx systems, may also play roles in HDGF-induced ROS generation during HCC progression. Therefore, uncovering the interplay between HDGF and mitochondrial antioxidant systems in HCC progression will be important in future work.

In summary, the present study showed that the HDGF/NCL axis accelerated mitochondrial bioenergetic activity and caused excess ROS generation that resulted in liver tumorigenesis. Our findings provide a fundamental mechanism underlying the regulatory effect of HDGF on mitochondrial energy metabolism in hepatocellular carcinoma.

## Experimental procedures

### Cell culture

Human hepatoma SK-Hep-1 and Huh-7 cells were purchased from the American Type Culture Collection with validated short tandem repeats-PCR profile and cultured in Dulbecco's modified Eagle's medium (DMEM) (GE HealthCare HyClone) containing with 10% fetal calf serum (GIBCO BRL), antibiotics (100 IU/ml penicillin and 100 μg/ml streptomycin) and L-glutamine (2 mM, Thermo Fisher Scientific). Rat Novikoff hepatoma N1-S1 cells were cultured in RPMI 1640 medium (GE HealthCare HyClone) supplemented with 10% calf serum (Hyclone), antibiotics, and L-glutamine. All the cell lines were incubated at humidified conditions with 95% air and 37 ˚C humidified incubator with 5% CO_2_. All the cell lines in this study were tested for *mycoplasma*. The cell lines with free contamination of *mycoplasma* were subjected to consequently experiments.

### HDGF protein, HDGF antibody, and adenovirus vectors

rHDGF and anti-HDGF antibodies were generated as previously described (54). The recombinant adenoviruses containing green fluorescent protein (Ad-GFP), HDGF complementary DNA (Ad-HDGF), and HDGF short hairpin RNA (Ad-shHDGF) were prepared as previously described (15). Both NCL and SOD2 knockdown stable clones were generated using lentivirus-mediated gene transferring system. Two SOD2 shRNA plasmids (shSOD2 #1 oligo sequence: CCGGGCACGCTTACTACCTTCAGTACTCGAGTACTGAAGGTAGTAAGCGTGCTTTTT; shSOD2 #2 oligo sequence: CCGGGTGGTGGTCATATCAATCATACCGAGTATG ATTGATATGACCACCACTTTTT.), and two NCL shRNA plasmids (shNCL#1 oligo sequence: CCG GCAAGGAAAGAAGACGAAGTTTCTCGAGAAACTTCGTCTTCTTTCCTTGTTTTTG; shNCL#2 oligo sequence: CCGGGCACTTGGAGTGGTGAATCAACTCGAGTTGATTCACCACTCCAAGTGCTTTTTG) as well as pLKO.1-puro-Luciferase shRNA control plasmid were purchased from the RNAi Consortium (TRC, Academia Sinica). To generate the shNCL and the shSOD2 lentiviruses, 293T cells were cotransfected with pLKO.1-shRNA, pCMV-ΔR8.9, and pMD.G vectors using lipofectamine 3000 reagent (Thermo Fisher Scientific) for overnight and then replaced with DMEM medium containing 10% fetal bovine serum. The lentivirus-contained medium was further harvested at 40 h and 64 h, respectively. For knockdown of either NCL or SOD2 in SK-Hep1 cells, cells were infected with the lentivirus-contained medium with polybrene (8 μg/ml, Sigma-Aldrich) for 24 h. The stable clones of either shNCL or shSOD2 were selected by using DMEM medium containing puromycin (5 μg/ml). The gene knockdown efficiency was evaluated by quantitative PCR and Western blotting.

### HDGF site-directed mutagenesis

Site-directed mutagenesis experiments to create HDGF mutants, including Ser103Ala (S103A) and Ser103Glu (S103E), were carried out according to the manufacturer’s directions (Stratagene). Briefly, mismatched oligonucleotides were designed (S103A HDGF: Forward 5′-GCTTCCGGCTATCAGTCC CCTCAGAAAAAGAGCTGTGTG-3′ and Reverse, 5′-CACACAGCTCTTTTTCTGAGCGGACTGATA GCCGGAAGC-3’; and S103E HDGF: Forward 5′-GCTTCCGGCTATCAGTCCGAGCAGAAAAAGA GCTGTGTG-3′ and Reverse, 5′-CACACAGCTCTTTTTCTGCTCGGACTGATAGCCGGAAGC-3′) to construct and generate HDGF mutants in the pET15b-HDGF plasmid using NdeI and BamHI restriction enzymes. Next, the two pET15b-HDGF mutants were transformed into *E. coli* strain BL21 (DE3, pLysS; Novagen) for protein generation and purification. The processes of protein generation and purification for these two rHDGF mutant proteins (S103A rHDGF and S103E rHDGF) are described as previously described (54).

### Established HCC animal models

Sprague-Dawley (SD) rats (male, 150 ± 50 g; n = 8) were purchased from the National Laboratory Animal Center. All animal care and treatment procedure were reviewed and approved by the Institutional Animal Care and Use Committee of National Sun Yat-sen University (IACUC No. 10822). For induction of the rat orthotopic hepatocellular carcinoma, N1-S1 cells (5 × 10^6^ cells in 100 μl of RPMI1640 medium per rat) were inoculated into liver parenchyma under ultrasound guidance that was described in our previous study (23). Additionally, HDGF KO mice (male, n = 6) were kindly provided by Professor Sebastian Franken (Germany) (55); WT C57BL/6 mice were purchased from BioLASCO.

### Immunohistochemistry analysis

The resected liver tissues were analyzed for HDGF expression profiles (14). Briefly, after deparaffinization, the resected tissues were blocked with 3% hydrogen peroxide for 10 min and retrieved the antigen epitopes by microwave in 10 mM citrate buffer for 30 min. The indicated antibodies were applied onto the sections and incubated at 4 °C overnight followed by repeated washing with PBS. Horseradish peroxidase/Fab polymer conjugate (Polymer detection system; Invitrogen/Zymed) was applied to the sections and the sections were incubated for 30 min. Finally, the sections were incubated with peroxidase substrate diaminobenzidine (1:20 dilution, Zymed) and counterstained with Gill’s hematoxylin before dehydration.

### *In situ* superoxide detection

The superoxide in frozen tissue sections were detected using a DHE (Invitrogen) staining as previously described (56). Briefly, the sections were incubated with DHE solution (5 μM) for 30 min at 37 °C in a humidified chamber and protected from light, and then the cell nuclei were stained with 4′,6-diamidino-2-phenylindole (DAPI) for 10 min. The fluorescent images represent superoxide generation were monitored and quantified using a fluorescent microscope (Leica Microsystems, Schweiz, AG–CH) and Image J (https://imagej.net/ij/) software (National Institutes of Health).

### Flow cytometry analysis of ROS generation

The intracellular ROS generation were measured by flow cytometry analysis using DHE and DCFH-DA (Invitrogen) staining, respectively. Cells (4 × 10^5^ cells) were treated with various concentrations of HDGF protein (1, 10, 100 ng/ml) for 4 h in serum-free DMEM medium, and these cells were incubated with DCFH-DA (10 *μ*M) or DHE (10 *μ*M) for 30 min at 37 °C. The ROS production was further analyzed using a CytoFLEX Flow Cytometer (Beckman Coulter, Inc) and CytExpert 2.0 software. In addition, to measure the mitochondrial superoxide anions, the MitoSox Red reagent (Invitrogen) was applied according to the manufacturer’s protocol. Briefly, cells (1 × 10^5^ cells/well) were seeded in 12-well culture plate contained with coverslips for overnight. Cells were treated with HDGF protein (10 ng/ml) for 4 h before incubation with MitoSox Red reagent (5 μM) for 10 min at 37 ˚C. After washing procedure and DAPI staining, the mitochondrial ROS expression profiles were further analyzed and recorded using the fluorescence microscope (Leica Microsystems, Schweiz, AG–CH).

### Cell proliferation assay

SK-Hep-1 cells were seeded at a 96-well culture plate (5 × 10^3^ cells/well) overnight. Cells were pretreated with or without NAC (5 mM; Sigma-Aldrich) for 1 h and then administrated with HDGF protein (1, 10, 100 ng/ml) in low serum (0.5%) medium for another 48 h. For detection of cell viability, cells were supplemented with 3-(4,5-dimethylthiazol-2-yl)-2,5-diphenyltetrazolium bromide (MTT, 0.5 mg/ml) and incubated for 2 h at 37 °C. The formazan in viable cells were dissolved with dimethyl sulfoxide (100 μl/well) and determined by reading optical densities in a microplate reader (Dynex Technologies, Inc) at an absorption wavelength of 570 nm.

### Invasion assay

Boyden chamber invasion assay has described as previous study (22). Briefly, the polycarbonate membrane (8 μm pore size, NeuroProbes, Inc) was coated with Matrigel (BD Bioscience) in advance. After pretreated with or without NAC (5 mM; Sigma-Aldrich) and MitoQ (1 μM; Sigma-Aldrich) for 1 h respectively, SK-Hep-1 cells were suspended and treated with HDGF protein (1, 10, 100 ng/ml) in serum-free DMEM medium. After treatment, the cell mixtures were loaded 50 μl into the upper chamber, while the lower chamber was supplemented with complete medium. The Boyden chamber was placed in a humidified CO_2_ incubator at 37 °C for 8 h. The invaded cells were fixed with methanol, stained with 10% Giemsa solution (Merck) and finally counted and recorded using inverted microscopy. The data were presented as the averages of triplicate experiments.

### Mitochondrial bioenergetics analysis

The OCR and ECAR in hepatoma cells were analyzed using a Seahorse XF24 extracellular flux analyzer (Seahorse Bioscience Inc) (57). Data were compared between the OCR (pmol/min/2 × 10^4^ cells) and the ECAR (mpH/min/2 × 10^4^ cells). Initially, cells were seeded in Seahorse cell culture 24-well plates (2 × 10^4^ cells/well) for overnight and then treated with HDGF protein (0–100 ng/ml) for 4 h. For anti-NCL antibody neutralization, cells were pretreated with NCL antibody (10 μg/ml, Santa Cruz Biotechnology) for 1 h and then incubated with rHDGF (10 ng/ml) for another 4 h. After wash with sodium bicarbonate-free DMEM medium, cells were refreshed with 675 μl of medium for further examination. The basic OCR was measured for four times and plotted as a function of the cells under basal conditions, the inhibitors including of oligomycin (1 μM), carbonyl cyanide-p-trifluoromethoxyphenylhydrazone (0.5 μM), and rotenone (1 μM) were added sequentially before experimental analysis. At the end of recording, cells were collected and counted using a trypan blue exclusion assay. The OCR and ECAR values were calculated after normalization with the number of cells.

### Measurement of ATP expression levels

The cellular ATP concentrations were measured using the ATP Bioluminescence Assay Kit CLS II (Roche) according to the manufacturer’s instructions. Briefly, after treatment with rHDGF for 4 h, cells (4 × 10^5^) were collected and resuspended in 200 μl of ATP assay dilution buffer (100 mM Tris, 4 mM EDTA, pH 7.5). Samples were boiled for 2 min at 100 °C and centrifuged for 5 min at 1000 g. Supernatants were collected and 50 μl samples were analyzed by Orion II microplate luminometer (Titertek-Berthold). The amount of ATP production was determined from a standard curve constructed with 10 to 100 pmol ATP.

### Mitochondrial staining

Cells were grown on glass coverslips for 16 h. After HDGF protein treatment for 4 h, cells were stained with MitoTracker Orange CM-H_2_TMRos (Molecular Probes) for 15 min at 37 °C, and then cells were fixed with 4% (v/v) paraformaldehyde for 5 min and permeabilized with PBS containing 0.1% (v/v) Triton X-100 and 2% (v/v) bovine serum albumin at room temperature for 5 min. Fixed Cells were probed with Alexa Fluor 488 phalloidin (Molecular Probes) for 15 min at 37 °C in the dark and then the cell nuclei were stained with DAPI for 10 min. Labeled cells were visualized with a LSM510 (Carl Zeiss).

### Western blot analysis

Western blot assay was performed as described (22). Cells were treated with various concentrations of HDGF protein (1, 10, 100 ng/ml) for 4 h in serum free DMEM medium. The cell lysates (20 μg) were subjected to electrophoresis on 10 to 12% SDS-PAGE. Primary antibodies were applied to detect proteins expression included SOD1, SOD2 (Santa Cruz Biotechnology) and catalase (Sigma-Aldrich). β-actin (Sigma-Aldrich) was used as internal control.

### Statistical analysis

Data were presented as mean ± SD from indicated repeats of experiments. The statistical analysis was performed using GraphPad Prism 5.0 (https://www.graphpad.com/support/prism-5-updates/) software (GraphPad Software). A *p* value less than 0.05 was considered statistically significant. Unpaired *t* tests were used to compare two groups. One-way ANOVA and post hoc multiple comparison using the Tukey test were used to compare three or more independent groups.

## Data availability

All the data produced for this work are contained within the article and the supporting information.

## Supporting information

This article contains supporting information.

## Conflict of interest

The authors declare that they have no conflicts of interest with the contents of this article.
